# Impact of coupling topology upon noise robustness of small optical reservoirs

**DOI:** 10.1038/s41598-020-70775-8

**Published:** 2020-08-24

**Authors:** Tian-You Cheng, Ching-Chuan Liu, Da-Ya Jhou, Chii-Chang Chen

**Affiliations:** Department of Optics and Photonics, National Central University, Jhongli, 32001 Taiwan

**Keywords:** Computational science, Optics and photonics

## Abstract

In this work, we perform the numerical investigation of the performance of the small optical reservoir computing (RC) systems with four neurons using the commercial software for optical fiber communication system. The small optical RC system consists of the components of the optical fiber communication. The nonlinear function which is required in RC is provided by the erbium-doped optical fiber amplifiers (EDFA). We demonstrate that the EDFA should be operated in the saturated or non-linear regime to obtain a better performance of the small optical RC system. The performance of the small optical RC systems for different topological neuron structures is investigated. The results show that the interconnection between the neurons could offer a better performance than the systems without interconnection between the neurons. Moreover, the input signals with different noise levels are launched into the systems. The results show that the small optical RC system can classify the noisy input optical waveforms even when the signal-to-noise ratio is as low as − 2.55 dB.

## Introduction

Recurrent neural network (RNN) is a brain-inspired computing for the information processing. Due to the non-linear function provided by the neurons, the input data can be classified in a higher-dimensional space^1^. RNN has been a very promising tool to deal with the time-dependent information^2,3^ including the speech recognition^4^, construction of gene regulatory network^5^, predicting protein structure and function^6^, and language model^7^, etc. Unfortunately, for RNN, the long training time and the numerous parameters to be optimized are required^8^. Therefore, RNN is not suitable for the applications where the short training time is necessary.

Based on RNN, the reservoir computing (RC)^9^ is a method in which the optimization is trained simply by the pseudo-inverse matrix method^10^. The time-consuming problem for the optimization using RNN may be alleviated. Many nonlinear functions such as Mackey–Glass oscillator^11^, tanh^8^, sinusoidal^12^, sigmoid^13^, and chaotic function^14^ can be used in the neurons. Several applications using RC have been demonstrated including speech/image recognition^15,16^, autonomous robots^17^, optical signal processing^18^, temporal information processing^19^, popularity prediction^20^, wind power ramp events predition^21^, attack detection of smart grids with wind power generators^22^, uncued brain-computer interface^23^, marking epileptic seizures on the intra-cranial electroencephalogram of rats^24^, non-linear time-series data analysis^25^, real-time audio processing^26^, real-time detection of epileptic seizures in animal models^27^, and noisy image recognition^28^, etc. Recently, we have also reported the deep learning of reservoir computing to predict the rainfall in Taiwan which is quite difficult to model theoretically in atmospheric science^29^.

The optical RC system has been experimentally demonstrated to be a candidate to perform the calculation in optics^30^. The nonlinear function required for the RC has been implemented by microring resonators^31–33^, birefringent interferometer^34^, LiNbO_3_ Mach–Zehnder interferometer^25,35^, semiconductor optical amplifiers (SOA)^36^, semiconductor saturable absorber mirror (SESAM)^37^. In the output layer, a LiNbO_3_ Mach–Zehnder intensity modulator and a balanced photodiode have been used to obtain the readout matrix^38^. However, the optical-electrical and the electrical-optical conversions are adopted to obtain the non-linear function in the loop in some of the systems^33,34^. These conversions could limit the calculation speed of the systems. Recently, we have also proposed a novel optical RC system to obtain the high-speed computing for the application of the input signal recognition^39^. In addition to the RC algorithm, the backpropagation has also been recently investigated the feasibility to be realized in integrated optics^40^. A photonic logical XOR gate may be achieved using this method.

In Refs.^12,18^, a non-trivial classification task is applied in the optical RC system using amplifiers and semiconductor optical amplifiers (SOA), respectively, to recognize the rectangular and triangular waveforms. In Ref.^18^, 16 SOAs are used to obtain the error rate lower than 0.1. In this work, we perform the numerical investigation of the performance of the small RC system formed by optical fibers using the commercial software for optical fiber communication system. The nonlinear function is obtained by the erbium-doped fiber amplifier (EDFA). No optical-electrical or electrical-optical conversion is required in the system. The performance of RC has been reported to be dependent on the interconnection topology^18,24,41^. The neurons in the reservoir should be randomly connected to obtain a high performance^8^. In this study, only four optical neurons are used forming a relatively small reservoir layer to investigate the fundamental properties from the small optical RC system. The performance of the small optical RC systems with different topological interconnection between the neurons is investigated. Moreover, we also study the performance of the small optical RC system with noisy input optical signals. The results show that the input signals with heavy noise can still be recognized.

## Principle of reservoir computing

**Figure 1:**
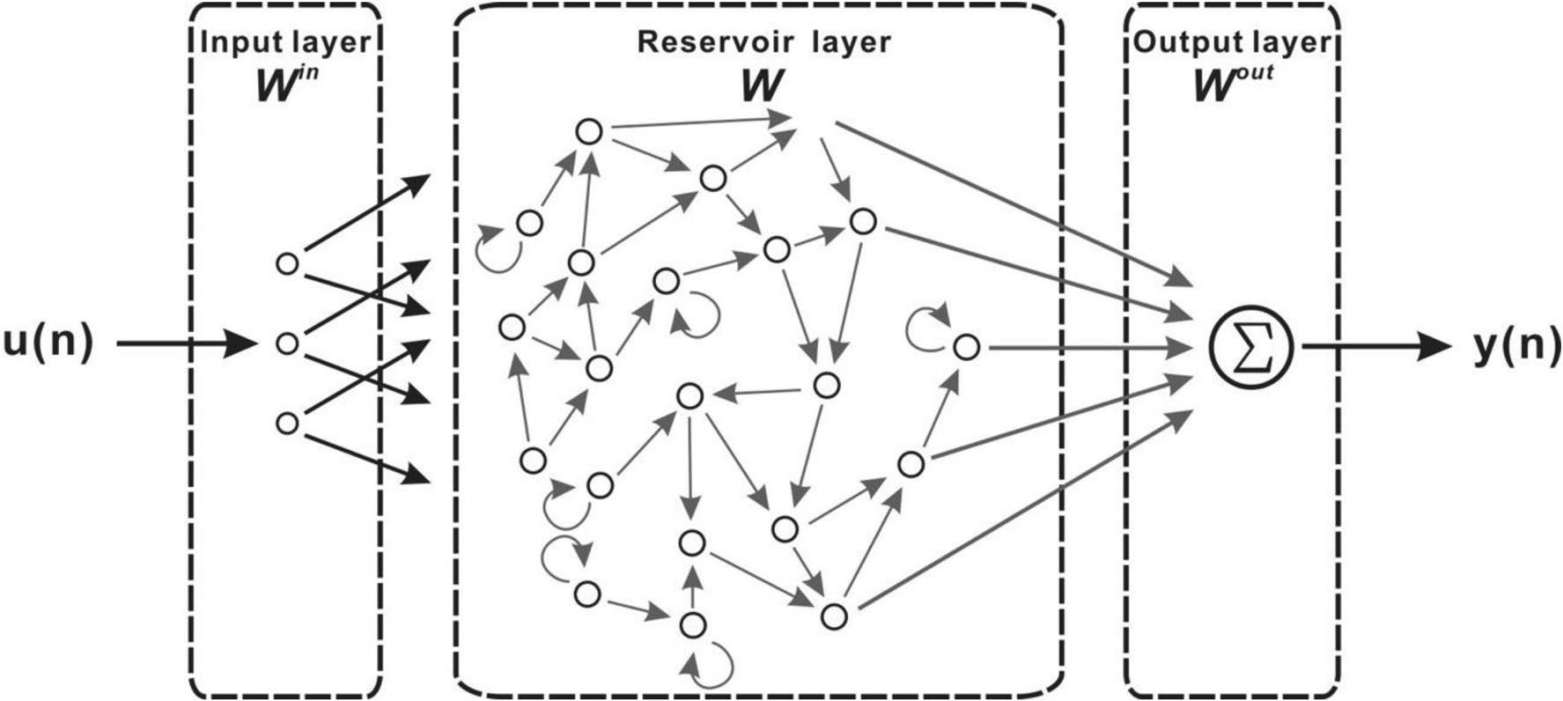
Schematic of the reservoir computing system.

The RC system is composed of the input, reservoir and output layers. The corresponding matrices are the input weight matrix ${W}^{in}$, the interconnection matrix $W$, and the readout matrix ${W}^{out}$, respectively^9^. Figure 1 shows the schematic of the RC system. ${W}^{in}$ of the input layer is used to scale the size of the input data to the size of the reservoir. The learning is completed in a single pass through training data in the reservoir layer. The connection between the optical neurons can be described by W. The optimal readout matrix ${W}^{out}$ in the output layer is used to convert the result of reservoir to the output of the RC system.

The neurons in the reservoir of the RC consist of the temporally internal states $x\left(n\right)$ which are perturbed by temporally external input *u*(n) in discrete time. Therefore, $x\left(n\right)$ is updated with time. The neuron can be described as a function of the current input and its previous calculation result which can be expressed by^9^

1 $$x\left(n\right)=f\left({{\varvec{W}}}^{in}\left[u\left(n\right)\right],{\varvec{W}}x(n-1)\right)$$

The function *f* is the nonlinear function of the neuron. The tanh() function is usually used. ${W}^{in}$ is the input weight matrix. $W$ is the interconnection matrix. The network output $y\left(n\right)$ is given by

2 $$y\left(n\right)={{\varvec{W}}}^{out}\left[x\left(n\right)\right]$$

where ${W}^{out}$ is the readout matrix. By collecting the data [*x(n)*] and the training target data, the readout matrix ${W}^{out}$ can be obtained by the pseudo-inverse matrix method^10^. The normalized root mean square error (NRMSE) is used to evaluate the difference between the theoretical output and the system output, given by^3^

3 $$ NRMSE = \frac{{\sqrt {\frac{{\mathop \sum \nolimits_{i = 1}^{N} \left( {Y^{\prime} - Y} \right)^{2} }}{N}} }}{{\left( {max\left( {Y^{\prime}} \right) - min\left( {Y^{\prime}} \right)} \right)}} $$

where ${Y}^{^{\prime}}$ and *Y* are the theoretical output and the system output of RC, respectively. *N* is the number of the evaluated samples. $max\left({Y}^{^{\prime}}\right)$ and $min\left({Y}^{^{\prime}}\right)$ represent the maximum and minimum values of ${Y}^{^{\prime}}$, respectively. When the system output of RC is close to the target output, the NRMSE approaches 0.

## Small optical RC system

### Input optical signals

**Figure 2:**
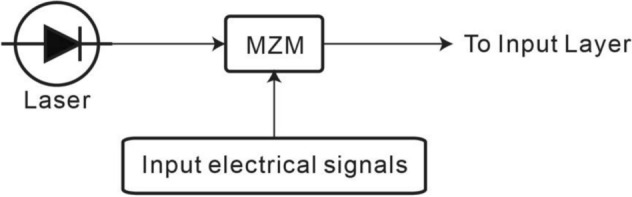
Generation of input optical signals. A laser at the wavelength of 1550 nm is chosen to serve as the light source. The Mach–Zehnder modulator is driven by the time-dependent input electrical signals, thereby producing the time-dependent optical signals. The optical signal is launched to the input layer.

Figure 2 shows the input signal generation of the small optical RC system. A laser emitting at the wavelength of 1,550 nm serves as the light source. The power of the laser is varied from 0.5 to 15 W to investigate the performance of the small optical RC system. Although the power of laser light above 1 W might induce the nonlinear effect in the optical fibers, in this work, we ignore the non-linear effect to investigate the performance of the small optical RC system. The light is launched into the Mach–Zehnder modulator (MZM) on which the input electrical signals are applied to produce the modulated optical signals.

### Optical neuron

**Figure 3:**
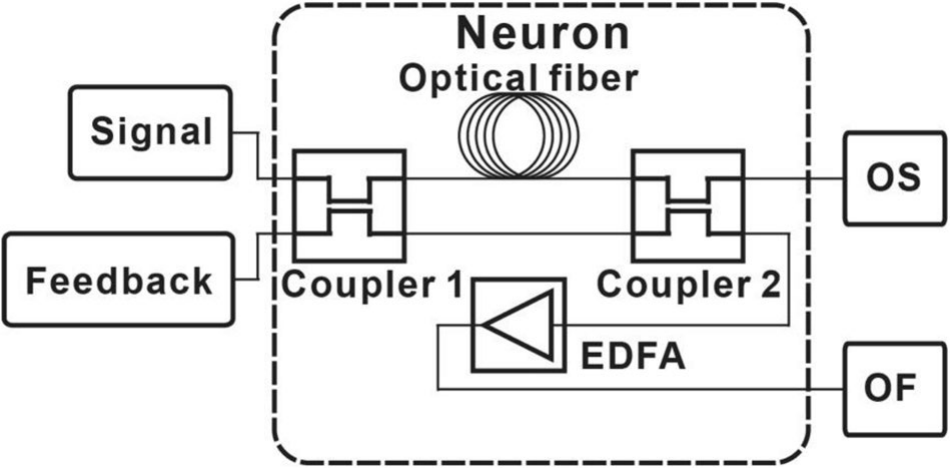
Structure of optical neuron consists of an EDFA, two directional couplers, and an optical fiber that acts as delay line. The length of the optical fiber is 0.49 m or 0.247 m for the parallel and serial structures, respectively.

The optical neuron is depicted in Fig. 3. It consists of an optical fiber, two directional couplers, and an EDFA. The short term memory of the optical neuron is obtained by the optical fiber. Therefore, the neuron can remember the previous input^3,37^. The nonlinear Kerr effect can be induced by the fiber-ring cavity. The roundtrip length of the fiber-ring cavity is 10 m^42^. The single mode with the length of 0.49 m and 0.247 m optical fiber was chosen for parallel and serial structure, respectively. The length of the optical fiber is much shorter than 10 m. Therefore, the nonlinear effect in the optical fiber is set to be 0. The coupling ratio of the directional couplers is 50%^43^. The directional couplers 1 and 2 are connected by the optical fiber and direct connection, respectively. One of the outputs of the directional coupler 2 serves as the output of the optical neuron. The output of the optical neuron could be connected to the signal port of the other optical neuron. (OS as illustrated in Fig. 3). Another one is connected to the EDFA which provides the nonlinearity. The saturation power of the EDFA is chosen to be 25 dBm for the small optical RC systems. After amplified by the EDFA, the signal is regarded to be the output of the optical neurons. (OF as illustrated in Fig. 3). OF of the optical neuron could be connected back to the feedback port (Feedback as illustrated in Fig. 3) of the same optical neuron. The recurrence of the signals is achieved. In this case, there is no interconnection between optical neurons. OF of the optical neuron could also be connected to the feedback of the other optical neurons. The interconnection between the optical neurons can be achieved.

In this study, triangular and rectangular signals are launched into the small optical RC system. The period of each rectangular or triangular signal is chosen to be 5 ns corresponding to a bit rate of 200 MHz. The refractive index of the optical fiber is 1.4682. The purpose of the optical fiber is to provide a delay of a half period (2.5 ns) between the input and output of the reservoir layer. The length of the optical fiber for different neuron network structures in the reservoir layer is described in the following section.

### Small RC systems with four optical neurons

**Figure 4:**
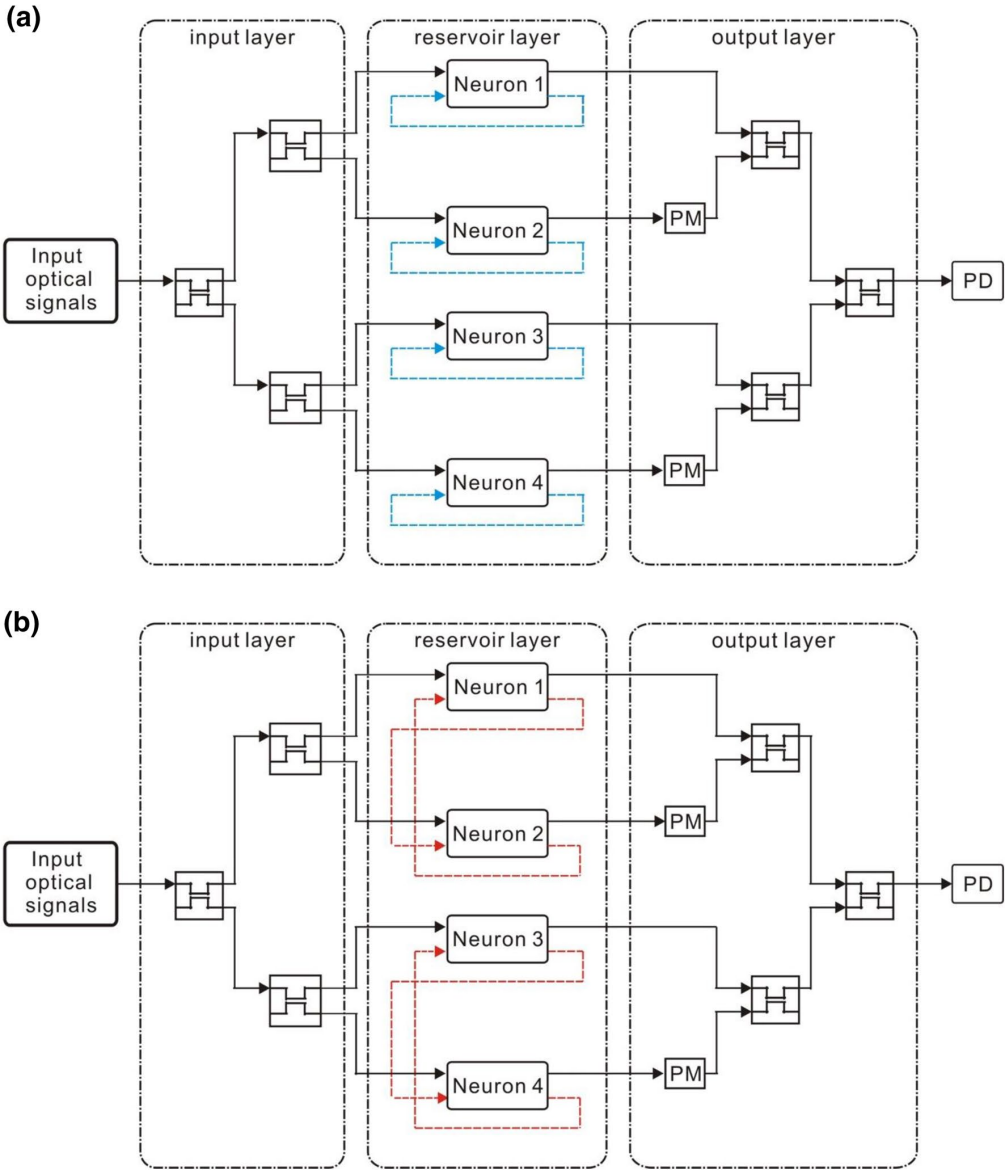
(**a**) Structure 1 and (**b**) Structure 2. PM and PD stand for the phase modulator and the photodetector, respectively. The blue dashed lines indicate that OF of the optical neurons is fed into Feedback of the same optical neurons. There is no interconnection between the optical neurons. The red dashed lines indicate that OF of the optical neurons is fed into Feedback of the other optical neurons. There is the interconnection between the optical neurons.

In this study, two types of the small optical RC systems with four optical neurons, parallel structure and serial structure, are investigated. The parallel structure is illustrated in Fig. 4. The input optical signals are launched into the input layer which consists of three directional couplers acting as the input weight matrix ${W}^{in}$. The coupling ratio of the directional couplers is both 55% which can be chosen randomly. An optical neuron is an element in the interconnection matrix W. The reservoir layer *W* consists of four optical neurons forming a 4 × 1 matrix. The length of the single mode fiber in the optical neurons is finely tuned to be 0.49 m to obtain a delay of a half period of the signal between the input and the output of the reservoir layer. In Fig. 4a (Structure 1), OF of each optical neuron is connected to its own Feedback [blue dashed lines in Fig. 4a]. There is no interconnection between the optical neurons. Structure 2 is depicted in Fig. 4b. OF signals of optical neuron 1 are fed into Feedback of optical neuron 2. OF signals of optical neuron 2 are fed into Feedback of optical neuron 1. There is the interconnection between the optical neurons 1 and 2. There are also the identical interconnections between the optical neurons 3 and 4 [red dashed lines in Fig. 4b]. The multiplication between the output signals of the optical neurons and the readout matrix ${W}^{out}$ is performed using the three directional couplers and the two phase modulators in the output layer.

**Figure 5:**
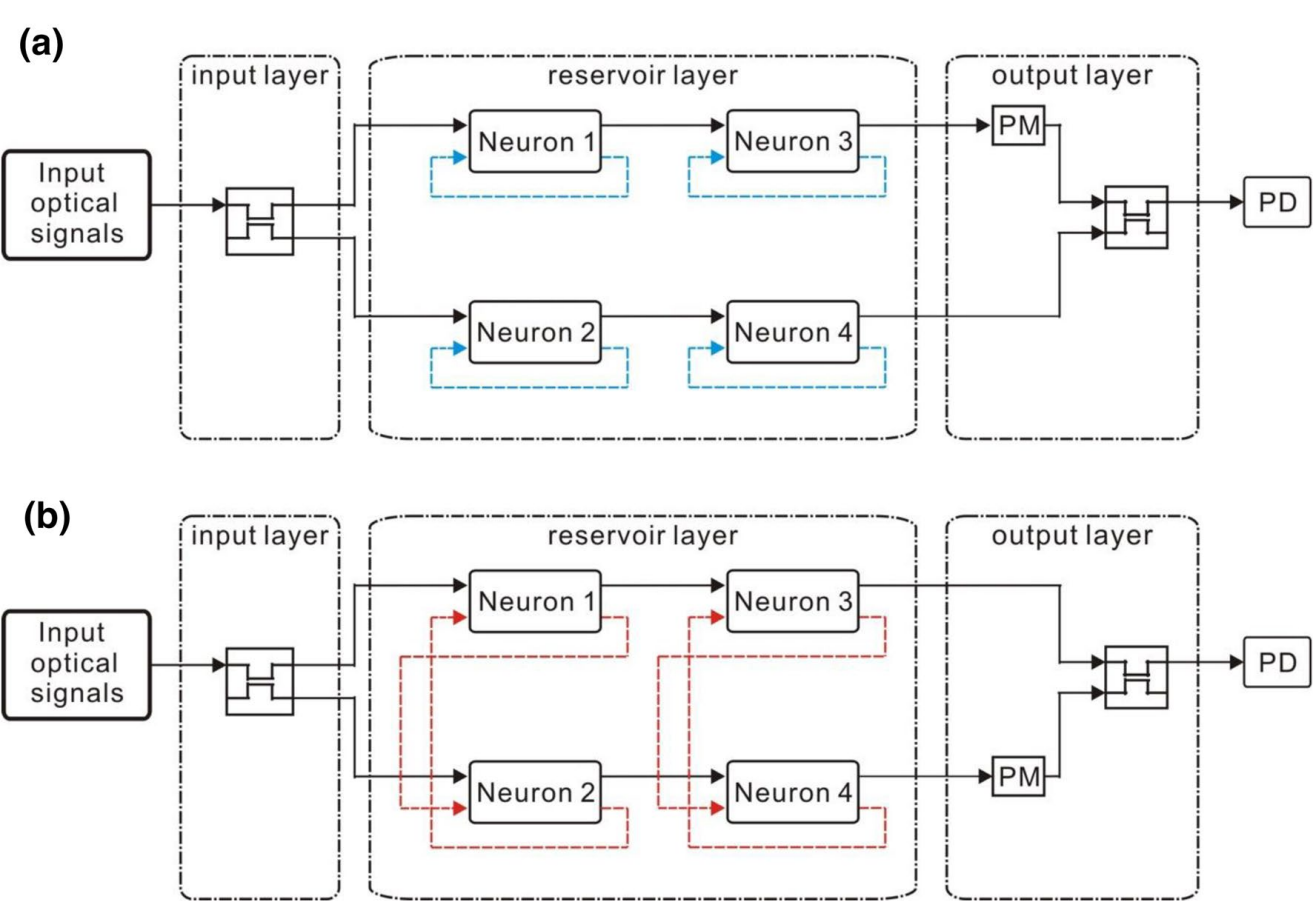
(**a**) Structure 3. The blue dashed lines indicate that OF of the optical neurons is connected with Feedback of the same optical neurons. There is no interconnection between the optical neurons. (**b**) Structure 4. The red dashed lines indicate that OF of the optical neurons is connected with Feedback of the other optical neurons. The optical neuron 1 and the optical neuron 3 have interconnection with the optical neuron 2 and the optical neuron 4, respectively. PM and PD stand for the phase modulator and the photodetector, respectively.

For the serial structure, two type of topologies are shown in Fig. 5. The input optical signals are launched into the input layer which consists of a directional coupler acting as the input weight matrix ${W}^{in}$. The coupling ratio is 55% which can be chosen randomly. An optical neuron is an element in the interconnection matrix *W*. The reservoir layer *W* consists of four optical neurons forming a 2 × 2 matrix. The length of the single mode fiber in the optical neurons is finely tuned to be 0.247 m to obtain a delay of half period of the signal between the input and the output of the reservoir layer. In Fig. 5a (Structure 3), each optical neuron’s OF signals are fed into its own Feedback.[blue dashed lines in Fig. 5a] There is no interconnection between the optical neurons. Namely, OF of each optical neuron is connected to its Feedback [blue dashed lines in Fig. 4a]. Structure 4 is depicted in Fig. 5b. OF signals of optical neuron 1 signals are fed into the Feedback of optical neuron 2. OF signals of optical neuron 2 are fed into Feedback of optical neuron 1 [red dashed lines in Fig. 5b]. The optical neurons 3 and 4 are connected identically [red dashed lines in Fig. 5b]. Therefore, there is the interconnection between the optical neurons. The multiplication between the output signals of the optical neurons and the readout matrix ${W}^{out}$ is performed using the directional coupler and the phase modulator in the output layer. By collecting the training signals from OS of the two optical neurons 3 and 4 as well as the training target data, the readout matrix ${W}^{out}$ can be obtained by the pseudo-inverse matrix method.

In the output layer, the coupling ratio of the directional coupler is chosen to be 50% to obtain the higher visibility of the optical interference. The phase modulator is finely tuned to obtain the lowest NRMSE. The optical signals are converted to the current by the photodetector.

OptSim, the commercial software, is widely used to simulate signal propagation in the optical communication systems. The signal propagates through the optical fiber, directional coupler, and EDFA can be simulated. Therefore, the small optical RC systems are simulated by OptSim. In this study, the length of the optical fiber is much shorter than the roundtrip length of the optical fiber-ring cavity^42^. The four types of the small optical RC systems in which the nonlinear function is obtained by the EDFA, were also used to analyze the noise. Therefore, the nonlinear effect of fiber, dispersion of fiber and amplifier noise of EDFA were ignoring.

In the summary of the simulation in this study, the coupling ratio of the directional couplers is 55% in the input layer. The length of the single mode fiber in the optical neuron is 0.49 m and 0.249 m in the parallel and serial structure, respectively. ${P}_{sat}$ of EDFA is chosen to be 25 dBm (0.316 W). In the output layer, the coupling ratio of the directional coupler is chosen to be 50%. The rest of the parameters of the simulation are shown in Table 1.

**Table 1:** The G_0_ of EDFA of Structures 1, 2, 3 and 4.

Structure	Parallel/serial	Interconnection	Neuron 1	Neuron 2	Neuron 3	Neuron 4
1	Parallel	No	3.07	2.2	1.33	2.2
2	Parallel	Yes	1.4	1	0.9	1
3	Serial	No	1.4	1	1.1	1
4	Serial	Yes	1.5	2	4	4.7

### Training

**Figure 6:**
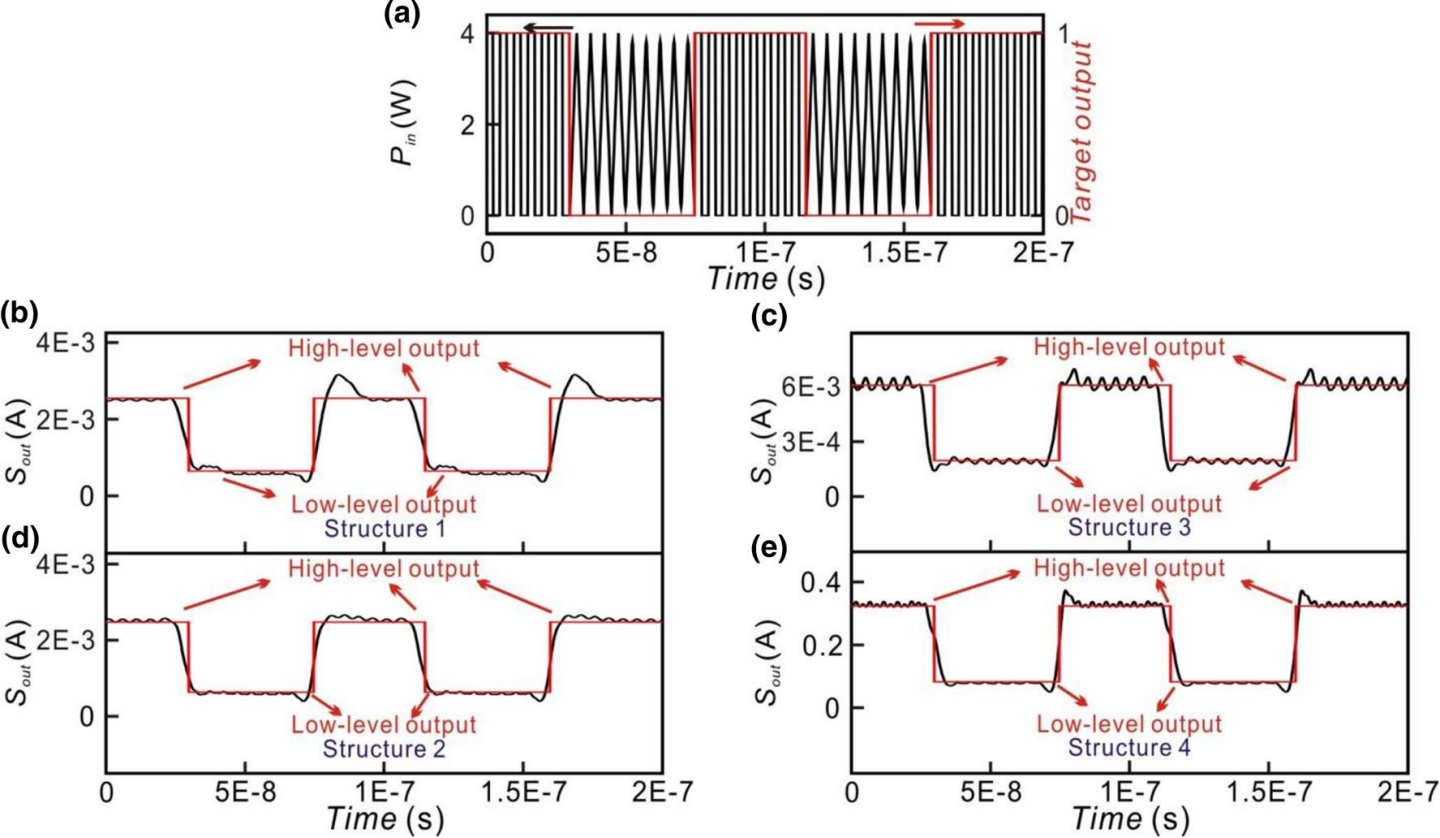
(**a**) Input optical signals (black lines) and target output (red lines) of small optical RC system. (**b**–**e**) represent the output signals of Structure 1, 2, 3 and 4, respectively.

To evaluate the performance of the small optical RC systems, the input optical signal as shown in Fig. 6a, which is composed of randomly arranged rectangular and triangular waveforms is generated by the laser and the Mach–Zehnder modulator. The input optical signals are modulated between 0 and 4 W at 200 MHz. The corresponding target outputs of the small optical RC system are 1 (high-level output) and 0 (low-level output) for the rectangular and triangular waveforms, respectively, as shown the red line in Fig. 6a. The outputs of the optical neurons of the reservoir layer are first collected. ${W}^{out}$ is obtained by the pseudo-inverse matrix method as well as the outputs of the optical neurons of the reservoir layer and the target output. Some of the elements of ${W}^{out}$ are negative. It indicates that the destructive interference between the outputs of the optical neurons is required in the directional couplers of the output layer. This can be achieved by connecting a phase modulator (PM) between the optical neurons and the directional couplers in the output layer as illustrated in Figs. 4 and 5. The optical delay of the phase modulators is tuned to obtain the lowest NRMSE.

In the parallel structure, we obtain the corresponding ${W}^{out}$ for Structures 1 and 2 $$\left[\begin{array}{cc}\begin{array}{cc}93.04& -94.37\end{array}& \begin{array}{cc}95.16& -94.37\end{array}\end{array}\right]$$ and $$\left[\begin{array}{cc}\begin{array}{cc}12.08& -37.12\end{array}& \begin{array}{cc}34.24& -10.66\end{array}\end{array}\right]$$, respectively. In both ${W}^{out}$ matrices of Structure 1 and Structure 2, the sign of the second and the fourth elements are negative. As shown in Fig. 4, the PMs have connected the optical neuron and directional couplers in the output layer to achieve destructive interference between the outputs of the optical neurons. The optical delay of the phase modulators is scanned from 0° to 180° to obtain the lowest NRMSE.

In the serial structures, the corresponding ${W}^{out}$ for Structure 3 and 4 are $$\left[\begin{array}{cc}-1.98& 2.95\end{array}\right]$$ and $$\left[\begin{array}{cc}3.12& -1.95\end{array}\right]$$, respectively. The signs of the two elements of both the ${W}^{out}$ matrices are opposite indicating that the destructive interference between the outputs of the two optical neurons is required in the directional coupler of the output layer. This is achieved by connecting a PM between neuron 1 and the directional coupler in the output layer. The optical delay of the phase modulators is scanned from 0° to 180° to obtain the lowest NRMSE.

## Results and discussion

The simulation results of Structures 1, 2, 3, and 4 are shown in Fig. 6b–e, respectively. Black and red lines represent the output signals of the small optical RC system and the target output signals, respectively. We can observe that the high-level and low-level outputs can be obtained when the input optical signals are rectangular and triangular waveforms, respectively. The result shows that the small optical RC system can classify the waveforms of the input optical signals.

**Figure 7:**
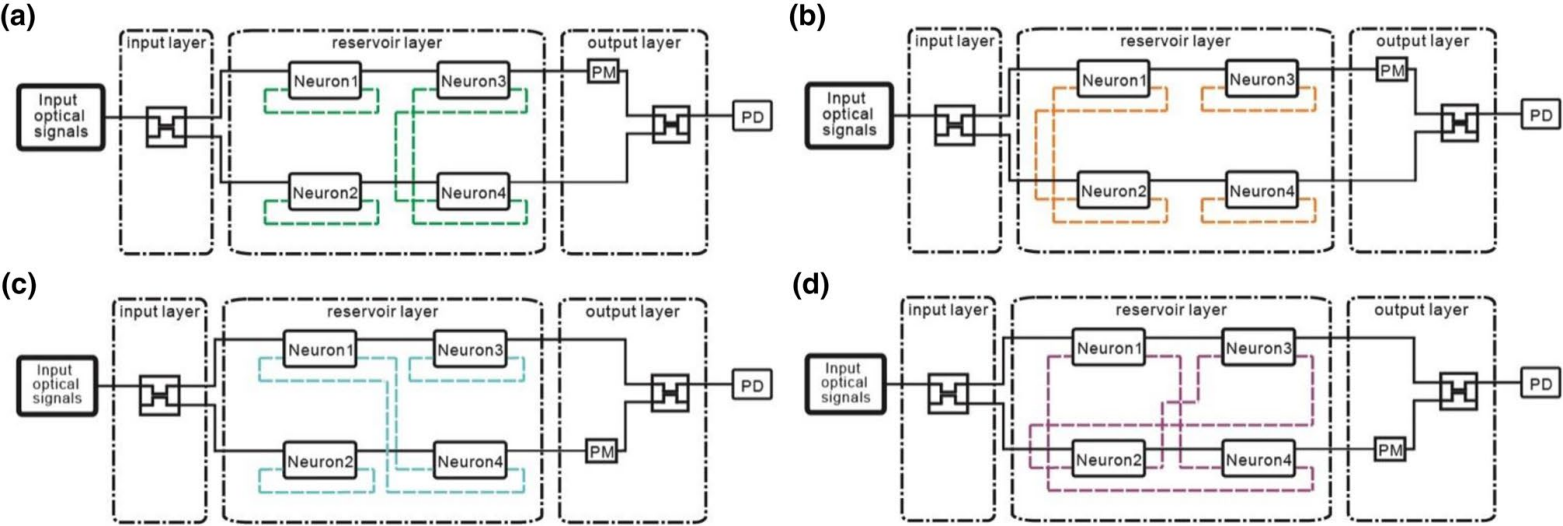
(**a**) Structure 5. OF of the optical neurons 1 and 2 are connected with Feedback of the same optical neurons. OF of the optical neuron 3(4) is connected with Feedback of the optical neuron 4(3). (**b**) Structure 6. OF of the optical neuron 1(2) is connected with Feedback of the optical neuron 1(2). OF of the optical neurons 3 and 4 are connected with Feedback of the same optical neurons. (**c**) Structure 7. OF of the optical neuron 2 and 3 are connected with Feedback of the same optical neurons. OF of the optical neuron 1(4) is connected with Feedback of the optical neuron 4(1). (**d**) Structure 8. OF of the optical neuron 1(4) is connected with Feedback of the optical neuron 4(1). OF of the optical neuron 2(3) is connected with Feedback of the optical neuron 3(2). PM and PD stand for the phase modulator and the photodetector, respectively.

To evaluate the NRMSE of the small optical RC system, *max(Y′)* and *min(Y′)* in Eq. (3) are calculated by averaging the power of the signals for high-level and low-level outputs, respectively. Namely, the low level of the target output is averaging the power of the signals for low-level outputs. Therefore, the low level of the target output is not zero as shown in Fig. 6b–e. The NRMSE for Structures 1 and 2 (Fig. 6b,c) is 0.176 and 0.128, respectively. The NRMSE for Structures 3 and 4 (Fig. 6d,e) is 0.197 and 0.11, respectively. The other four types of topologies are shown in Fig. 7. The NRMSE of these structures is shown in Table 2. It is found that NRMSE depends on the connection topology indicating that the performance of the small optical RC system depends on the connection topology^41^. In the parallel structure, the NRMSE of Structure 2 is lower than Structure 1. In the serial structure, NRMSE of Structure 4, 5, 6, 7, 8 is lower than Structure 3. The NRMSE of the small optical RC system with interconnection is lower than that without interconnection. The results are consistent with the property of RC method in which the optical neurons in the reservoir should be randomly connected^4^. The NRMSE of Structure 2 and Structure 4 is the lowest in the small optical RC system with interconnection between the optical neurons. Therefore, Structure 2 and 4 are used to discuss the relation between the topology of structure and NRMSE.

**Table 2:** NRMSE of Structures 5, 6, 7, 8, 7 and 8 for the input optical powers of 4 W.

Structure	NRMSE@4 W
5	0.161
6	0.156
7	0.161
8	0.187

### Performance of small optical RC system for different operation regimes in EDFA

According to the property of RC method, the neuron should be activated by a nonlinear function to provide the capability to classify the input data into a higher-dimensional space^8^. In this study, the nonlinear function is provided by the EDFA in the optical neurons. The gain coefficient *G* of the gain model of EDFA in our simulation can be expressed as

4 $$\mathrm{G}=\frac{{\mathrm{G}}_{0}}{1+{\mathrm{G}}_{0}\frac{{\mathrm{P}}_{in}}{{\mathrm{P}}_{\mathrm{sat}}}}$$

where ${G}_{0}$ is the small signal gain. ${P}_{sat}$ is the saturation output power. ${P}_{in}$ is the input power. ${P}_{sat}$ of EDFA is chosen to be 25 dBm (0.316 W). ${G}_{0}$ of EDFA dependents on the structure.

**Figure 8:**
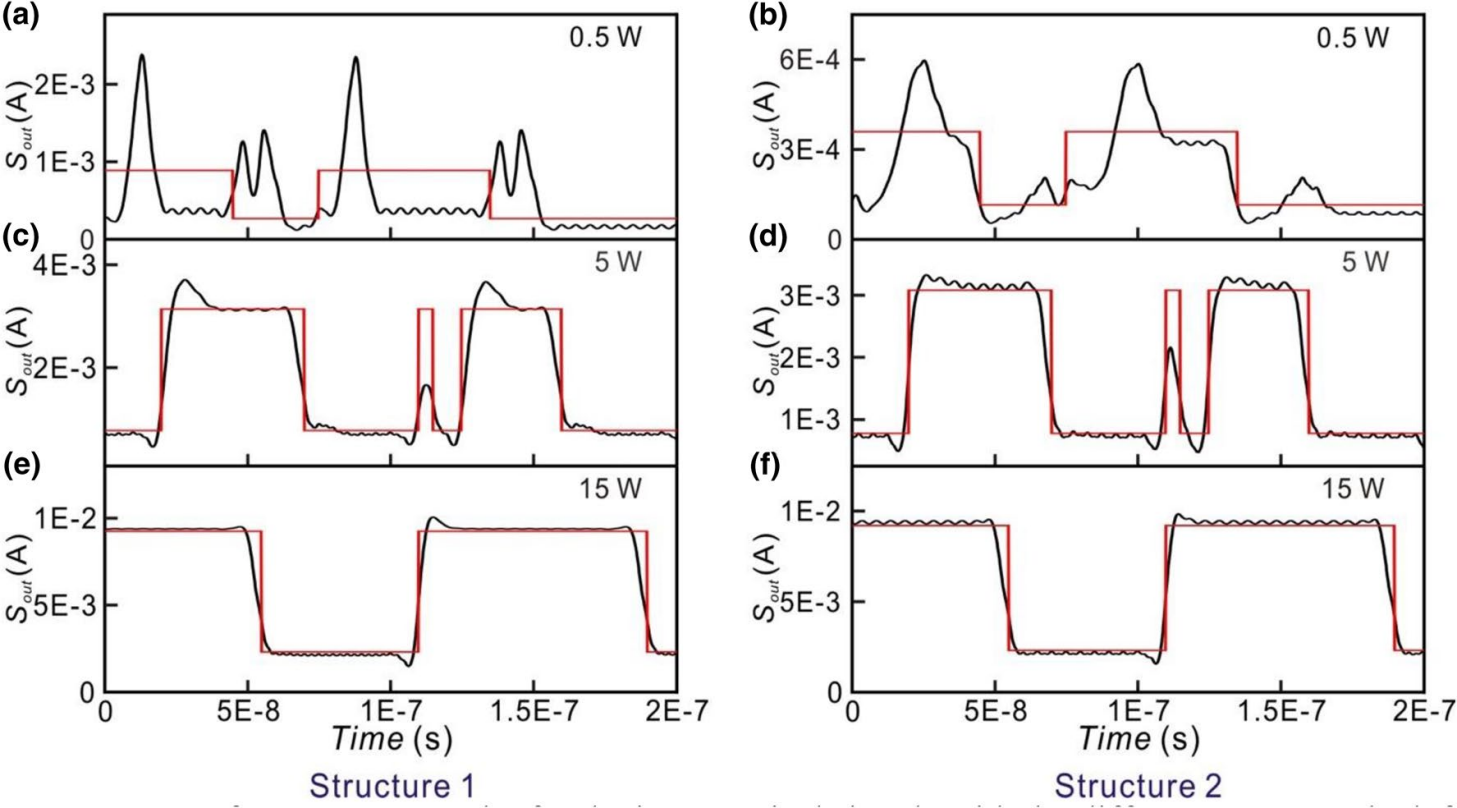
Outputs of Structures 1 and 2 for the input optical signals with the different powers. The left and right hand figures are the outputs of Structure 1 and Structure 2, respectively. From top to bottom: the power of the input optical signals is (**a**,**b**) 0.5 W (**c**,**d**) 5 W (**e**,**f**) 15 W. The red lines indicate the target output.

**Figure 9:**
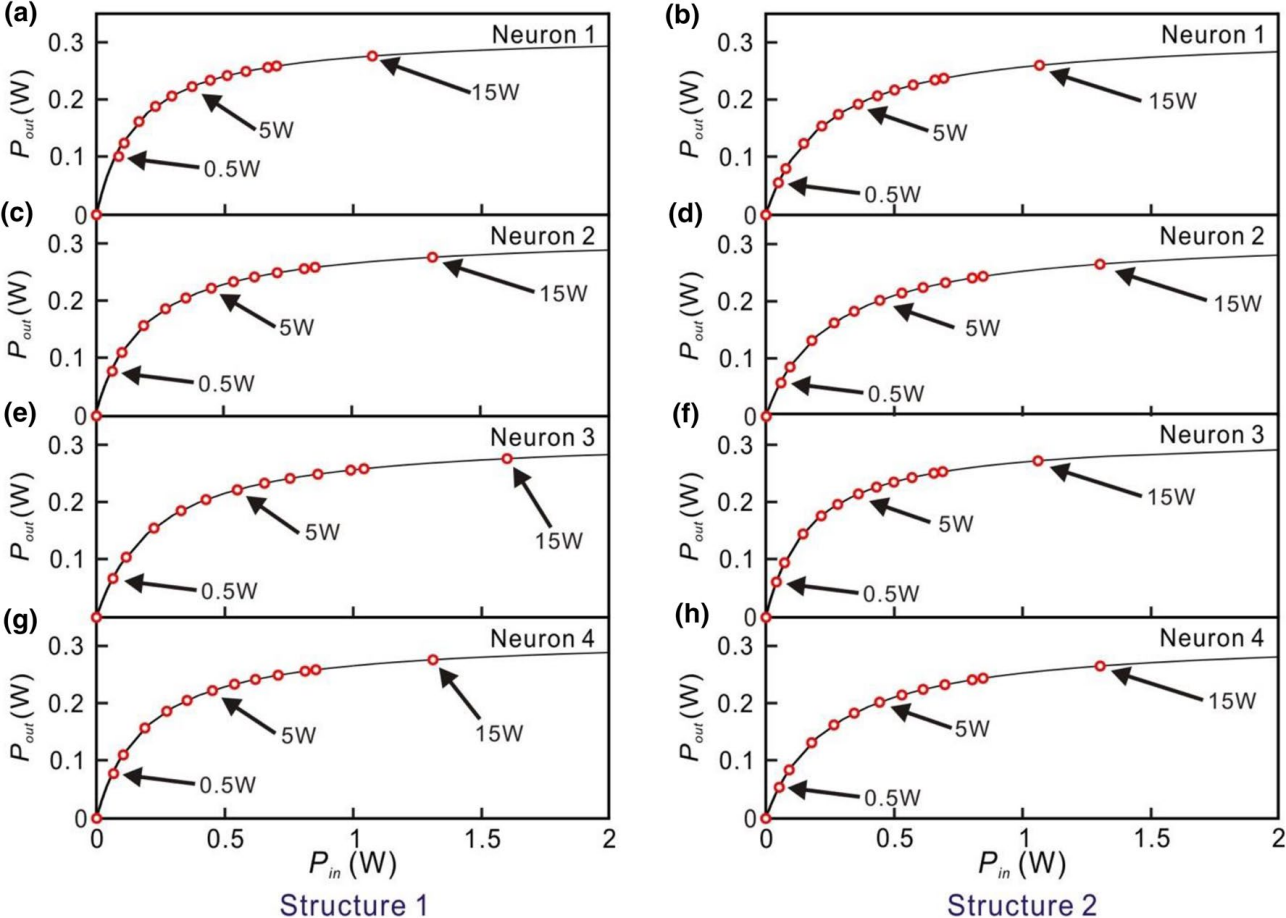
Average output power of the EDFA as the average input power of the EDFA is varied from 0 to 2 W (Psat = 0.316 W). The left hand figures are the results of Structure 1. From top to bottom: G0 is (**a**) 3.07, (**c**) 2.2, (**e**) 1.33, and (**g**) 2.2. The right hand figures are the results of Structure 2. From top to bottom: G0 is (**b**) 1.4, (**d**) 1, (**f**) 0.9, and (**h**) 1. The input power of the laser of the small optical RC system is 0 W, 0.5 W, 1 W, 2 W, 3 W, 4 W, 5 W, 6 W, 7 W, 8 W, 9 W, 10 W and 15 W. (red circles).

To study the performance of the small optical RC system for the different operation regimes in EDFA, the power of the input optical signal of the laser is varied from 0.5 to 15 W. The power of the modulated optical signal is divided by the couplers of the input layer. Since the coupling ratio of the couplers in the input layer is chosen randomly, the power received by each neuron is different. We analyze the input and output power of the EDFAs in each neuron for Structures 1 and 2 which are both in the parallel structure. The output signals of the small optical RC systems for the input optical signal of the laser with different powers (0.5 W, 5 W, 15 W) are shown in Fig. 8. The relations between the input power (horizontal axis) and output power (vertical axis) of the EDFAs for each neuron in Structures 1 and 2 are shown in Fig. 9. The corresponding powers (0.5 W, 5 W, 15 W) of the input optical signals of the laser are also indicated. When the power of the input optical signal of the laser is 0.5 W, the output signals of Structures 1 and 2 are shown in Fig. 8a,b, respectively. In this case, the EDFAs in the optical neurons operate in the linear regime as shown in Fig. 9. The corresponding NRMSEs of Structures 1 and 2 are 0.691 and 0.371, respectively as listed in Table 3. When the target output is low, some unwanted high output peaks appear as shown in Fig. 8a. When target output is high, some large peaks are higher than target output as shown in Fig. 8a. It indicates that the capability of the signal recognition of the RC system is very low.

**Table 3:** NRMSE of Structures 1, 2, 3 and 4 for the different input optical powers.

Structure	Parallel/serial	Interconnection	NRMSE@0.5 W	NRMSE@4 W	NRMSE@5 W	NRMSE@15 W
1	Parallel	No	0.691/Fig. 7a	0.176/Fig. 6b	0.149/Fig. 7c	0.118/Fig. 7e
2	Parallel	Yes	0.371/Fig. 7b	0.128/Fig. 6c	0.124/Fig. 7d	0.118/Fig. 7f
3	Serial	No	1.082/Fig. 9a	0.197/Fig. 6d	0.194/Fig. 9c	0.196/Fig. 9e
4	Serial	Yes	0.114/Fig. 9b	0.11/Fig. 6e	0.1/Fig. 9d	0.096/Fig. 9f

When the power of the input optical signal of the laser is 5 W, the EDFAs of optical neurons operate in the nonlinear regime as shown in Fig. 8. The output signals of Structures 1 and 2 are shown in Fig. 8c,d, respectively. The corresponding NRMSEs of Structures 1 and 2 are 0.149 and 0.124, respectively. When the power of input optical signals of the small optical RC system is 15 W where the EDFAs of the optical neurons operate in the saturated regime, the output signals of Structures 1 and 2 are shown in Fig. 8e,f, respectively. The corresponding NRMSE of both Structures 1 and 2 are 0.118. The results imply that the output signals (black lines) are close to target output (red lines) when the EDFAs of the optical neurons operate in the nonlinear and saturated regimes.

**Figure 10:**
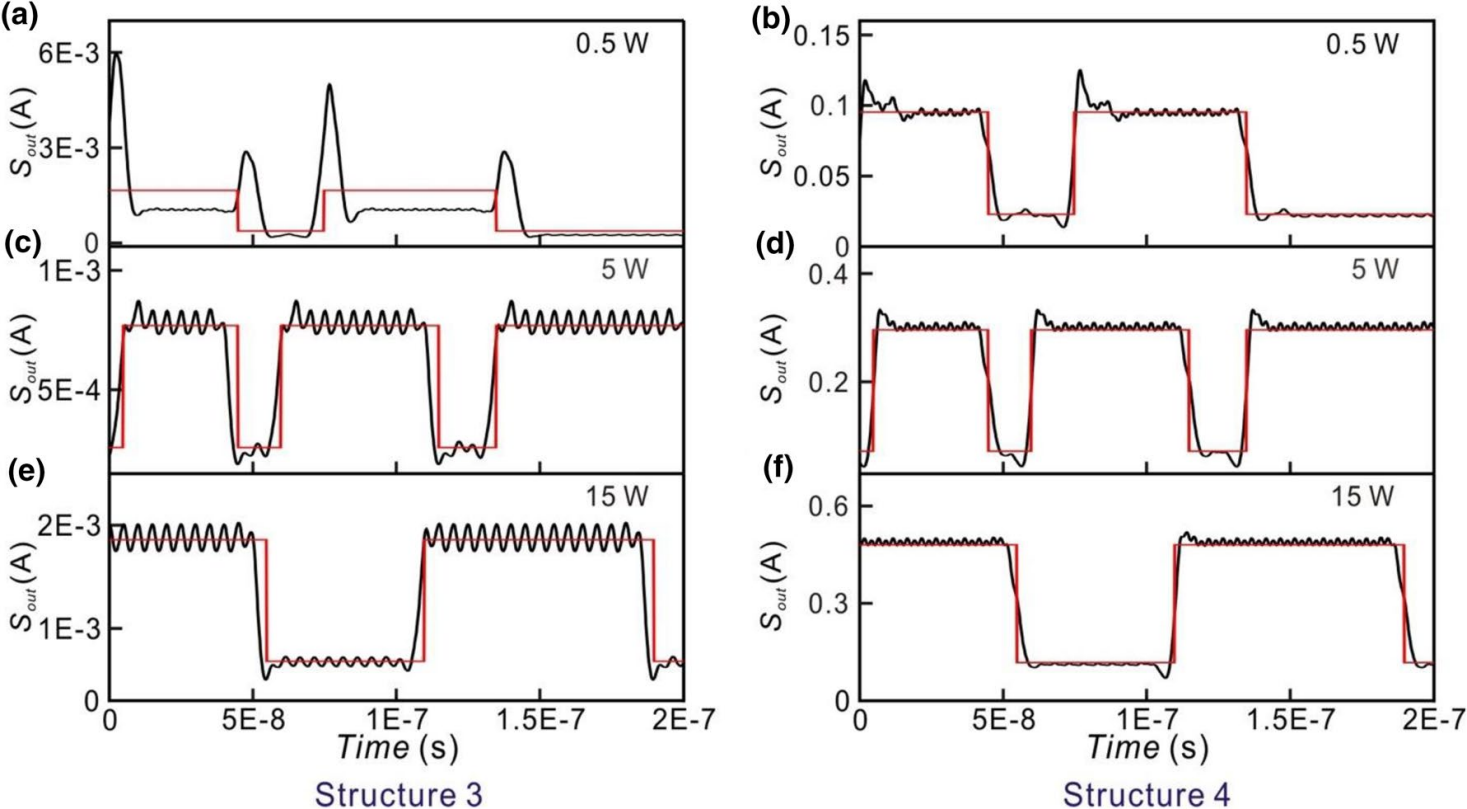
Outputs of Structure 3 and 4 for the input optical signals with the different powers. The left and right hand figures are the outputs of Structure 3 and Structure 4, respectively. From top to bottom: the power of the input optical signals is (**a**,**b**) 0.5 W (**c**,**d**) 5 W (**e**,**f**) 15 W. The red lines indicate the target output.

**Figure 11:**
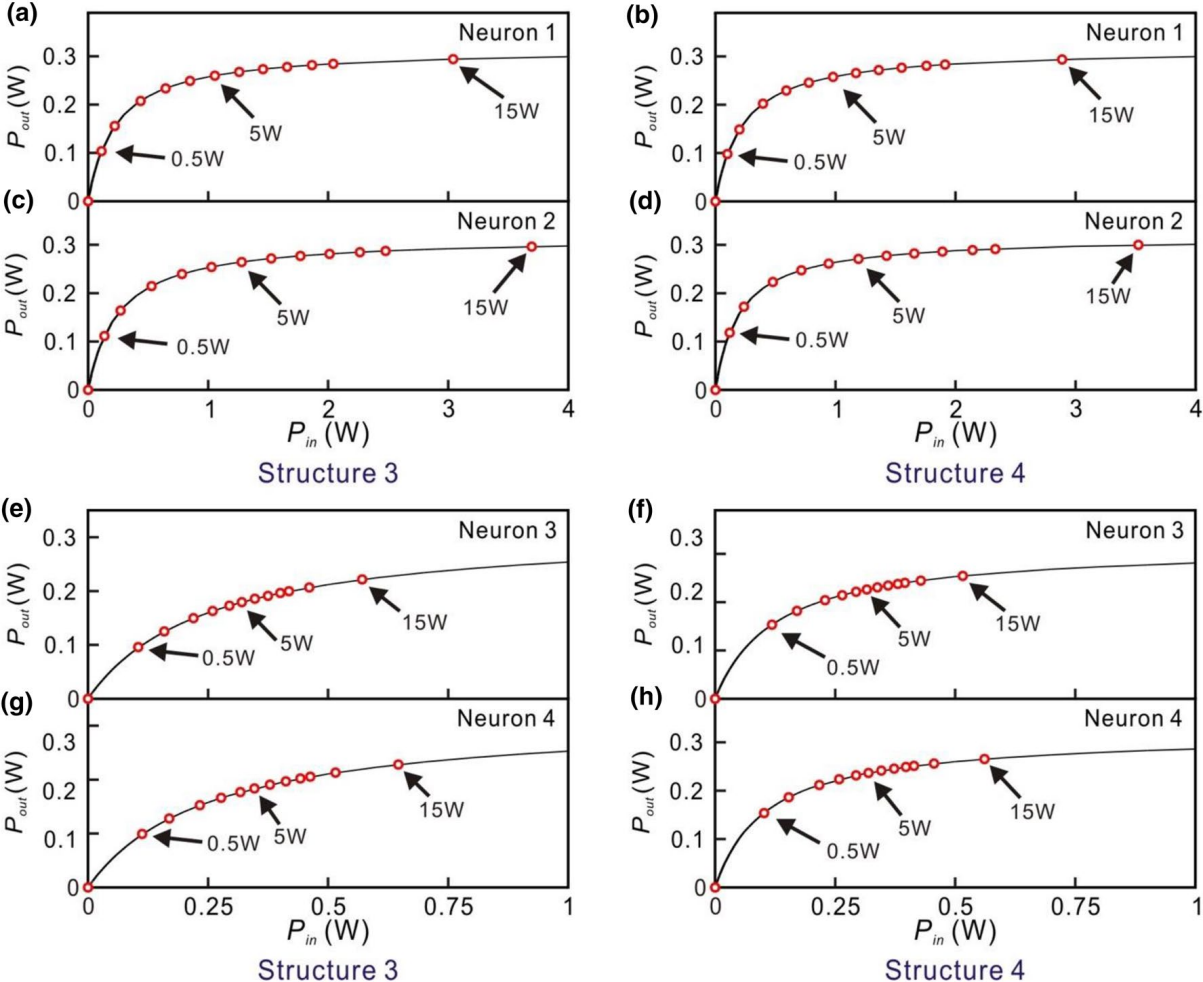
Average output power of the EDFA as the average input power of the EDFA is varied from 0 to (**a**,**b**, **e**,**f**) 4 W (**c**,**d**,**g**,**h**) 1 W. (Psat = 0.316 W) The left hand figures are the results of Structure 3. From top to bottom: G0 is (**a**) 1.4, (**c**) 1, (**e**) 1.1, and (**g**) 1. The right hand figures are the results of Structure 4. From top to bottom: G0 is (**b**) 1.5, (**d**) 2, (**f**) 4, and (**h**) 4.7. The input power of the laser of the small optical RC system is 0 W, 0.5 W, 1 W, 2 W, 3 W, 4 W, 5 W, 6 W, 7 W, 8 W, 9 W, 10 W, 15 W (red circles).

For Structures 3 and 4 which are in the serial structure, the output signals of the small optical RC systems for the input optical signal of the laser with the different powers are shown in Fig. 10. When the power of the input optical signal of the laser is 0.5 W, the output signals of Structures 3 and 4 are shown in Fig. 10a,b, respectively. When the target output is low, some unwanted high output peaks appear as shown in Fig. 10a. It indicates that the capability of the signal recognition of the RC system is very low. The corresponding NRMSEs of Structures 3 and 4 are as high as 1.082 and 0.114, respectively. In this case, the EDFAs in the optical neurons 1 and 2 in Structures 3 and 4 operate in the linear regime as shown in Fig. 11a–d. The EDFAs in the optical neurons 3 and 4 in Structures 3 and 4 operate in the nonlinear regime as shown in Fig. 11e–h. Figure 10c,d show the output signals of Structures 3 and 4, respectively, when the power of the input optical signal of the laser is 5 W. The corresponding NRMSEs of Structures 3 and 4 are 0.194 and 0.1, respectively. All of the EDFAs in the optical neurons operate in the nonlinear regime as shown in Fig. 11. When the power of the input optical signals of the laser is 15 W, the EDFAs of the optical neurons operate in the saturated regime. The output signals of Structures 3 and 4 are shown in Fig. 10e,f, respectively. The corresponding NRMSEs of Structures 3 and 4 are 0.196 and 0.096, respectively. The results show that the nonlinear or saturated operation of EDFA is favored to obtain the lower NRMSE.

**Figure 12:**
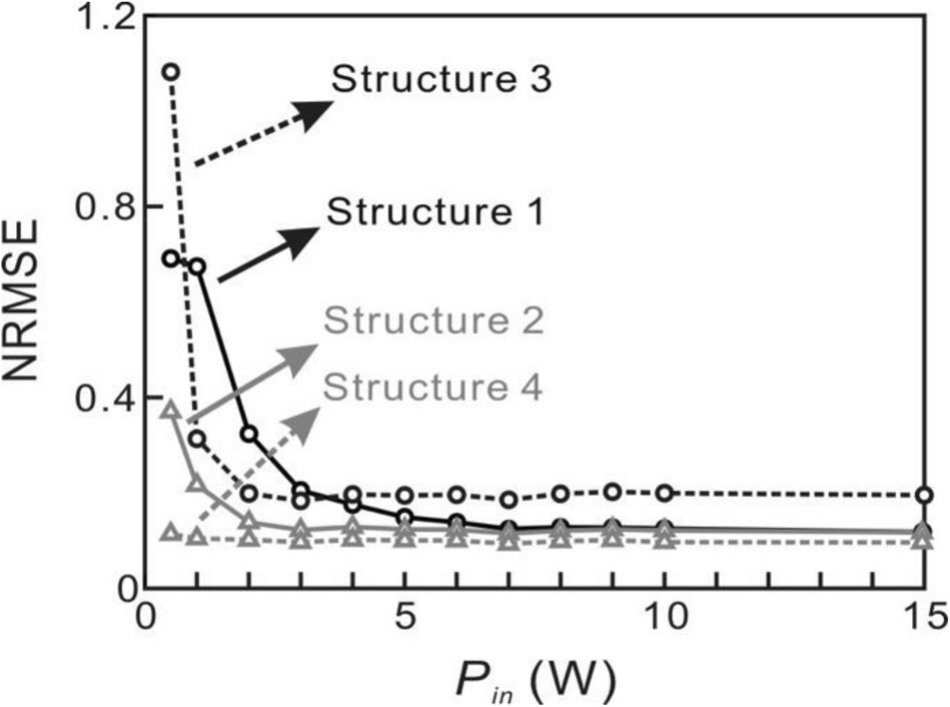
The NRMSE versus the power of the input optical signals. The solid lines and dashed lines represent parallel (Structure 1 and 2) and serial (Structure 3 and 4) structures, respectively. The black and gray lines indicate the small optical RC systems without interconnection (Structure 1 and 3) and with interconnection (Structure 2 and 4) between optical neurons, respectively.

The relation between NRMSE and the power of the input optical signals are depicted in Fig. 12. The power of the input optical signals of the small optical RC systems is varied from 0.5 to 15 W. In the parallel structures, when the power of the input optical signals of the small optical RC systems is 0.5 W where the EDFA in Structures 1 and 2 are checked to operate in the linear regime, the NRMSE of Structures 1 and 2 are 0.691 and 0.371, respectively. When the power of the input optical signals of the small optical RC systems is increasing from 1 to 3 W where the operation regime of EDFA varies from linear to nonlinear regime, the NRMSE decreases from 0.674 to 0.206 (black line) and 0.217–0.123 (gray line), respectively. When the power of the input optical signals of the small optical RC systems reaches 7 W where the EDFA is operating in the saturated regime, the NRMSE reaches the minimum. The NRMSE of Structures 1 and 2 is 0.124 and 0.115, respectively. As the power of the input optical signals of the small optical RC systems is higher than 7 W, the NRMSE is almost constant where the EDFA operates in the saturated regime.

In the serial structures, when the power of the input optical signal of the small optical RC systems is 0.5 W, the EDFA in the optical neurons 1 and 2 in Structures 3 and 4 operates in the linear regime. The EDFA in the optical neurons 3 and 4 in Structures 3 and 4 operates in the nonlinear regime. The corresponding NRMSE of Structures 3 and 4 are 1.082 and 0.114, respectively. When the power of the input optical signals of the small optical RC systems is increasing from 1 to 2 W, the operation regime of the EDFA in the optical neurons 1 and 2 in Structures 3 and 4 varies from linear to nonlinear regime. The NRMSE of Structures 3 and 4 decreases from 0.314 to 0.199 (black dashed line) and 0.104–0.102 (gray dashed line), respectively. When the power of the input optical signals of the small optical RC systems is higher than 3 W, the EDFAs operate in the saturated regime. The NRMSE reaches the minimum and is almost constant. The NRMSE of Structures 3 and 4 is 0.184 and 0.097, respectively. This result shows that the low NRMSE with the optical input power of 0.5 W in Structure 4 that nonlinear effect of optical fiber might not be significant.

Some major characteristics can be summarized from the results. First, the nonlinearity of the neurons is required to obtain the capability of signal recognition in the small optical RC system. Second, the minimum NRMSE can be obtained when the EDFA is operated in the saturated regime. Third, the NRMSE of Structures 2 and 4 are lower than Structures 1 and 3, respectively, for the same input optical power. This implies the fact that the optical neurons in the small reservoir should be randomly connected (with interconnections) to obtain a better performance. We can observe that the NRMSE of Structure 4 is the lowest and stays almost constant from 0.5 to 15 W. The results imply that the serial structure with interconnection between the optical neurons could be the best configuration.

In this study, the small optical RC system is also tested to recognize the waveforms with white noise. The power of the input optical signals of the small optical RC systems is 5 W and the power of white noise is varying from 0.5 to 9 W. The corresponding signal-to-noise ratio (SNR) is varied from 10 to − 2.55 dB. In past, this task should be done with signal recovery to reduce the noise and the signal recognition with neural networks, respectively. In this study, we show the performance of our small optical RC system to accomplish these two jobs. Structures 2 and 4 are used to investigate the performance of the small optical RC system.

The NRMSEs of Structures 2 and 4 for the input optical signals with the white noise of the different powers are listed in Table 4. We can observe that as the power of the white noise increases, the NRMSE increases. As the power of the white noise is less than 9 W (SNR = − 2.5 dB), the NRMSE is less than 0.5 indicating that the half input optical signals could still be recognized. In this signal recognition task, the NRMSE of Structure 2 and 4 are similar. The application could be the speech recognition in a noisy environment.

**Table 4:** NRMSE of Structures 2 and 4 for the input optical signals with the white noise of the different powers.

Power of white noise (W)	Structure 2	Structure 4
0	0.1239	0.1005
0.5	0.1743	0.1588
3	0.2974	0.3049
6	0.3992	0.4203
9	0.4601	0.4895

## Conclusion

The small optical reservoir computing system based on optical fiber communication system has been proposed. The optical neuron in the reservoir consists of optical fibers, directional couplers, and erbium-doped optical fiber amplifiers. The NRMSE of the small optical RC systems, which consist of four optical neurons, has been investigated. The optical neurons should be activated by a nonlinear function to obtain a better performance. The results show that the performance of the small optical RC system depends on the connection topology. The NRMSE of the RC systems with interconnection between the optical neurons is lower than that without interconnection between the optical neurons. The serial structure with interconnection between the optical neurons could be the best configuration. The noisy waveforms can be classified by the small optical RC systems even when the SNR is − 2.55 dB. The deep echo state networks have been reported recently^44^. By increasing the number of reservoirs, the error rate could be lower. Based on the results obtained in our study, the deep echo state networks could also be realized with the optical neurons. The more complex signal recognition task may be applied.
